# Knowledge of antibiotic resistance and antibiotic prescription practices among prescribers in the Brong Ahafo Region of Ghana; a cross-sectional study

**DOI:** 10.1186/s12913-017-2365-2

**Published:** 2017-06-20

**Authors:** Kwaku Poku Asante, Ellen Abrafi Boamah, Martha Ali Abdulai, Kwame Ohene Buabeng, Emmanuel Mahama, Francis Dzabeng, Edith Gavor, Edith Andrews Annan, Seth Owusu-Agyei, Martha Gyansa-Lutterodt, On behalf of the Ghana Antimicrobial Resistance Working Group

**Affiliations:** 1 Kintampo Health Research Centre, Ghana Health Service, Kintampo, Brong Ahafo Region, Ghana; 2 Faculty of Pharmacy & Pharmaceutical Services, College of Health Sciences, Kwame Nkrumah University of Science and Technology, Kumasi, Ghana; 3 Ghana National Drugs Programme, Ministry of Health, Accra, Ghana; 4 World Health Organization, Ghana Country Office, Accra, Ghana

**Keywords:** Antibiotic resistance, Prescribers, Knowledge, Prescription, Practices

## Abstract

**Background:**

Antibiotic resistance (ABR) has become a major public health challenge in most parts of the world including Ghana and is a major threat to gain in bacterial disease control. The role of prescribers in the control of antibiotics is identified as crucial in developing interventions to control ABR. To guide policy recommendations on ABR, a study was carried out among prescribers to identify gaps in their knowledge of ABR and to document their prescription practices.

**Method:**

A cross-sectional survey was conducted among prescribers from both public and private facilities in the Brong Ahafo Region of Ghana using both quantitative and qualitative methods in 2014.

**Results:**

Three hundred and seventy nine prescribers participated in the quantitative study and a subset of 33 participated in in-depth interviews. Majority (50.0%) of the prescribers interviewed were nurses. Most (51.0%) of the prescribers were located in hospitals. Knowledge of ABR was high among all the prescribers. About 80.0% percent of all prescribers agreed that the antibiotics that are currently used could lose its efficacy in future. There is no singular formal source of information on antibiotic resistance. The prescribers held a strong perception that antibiotic resistance is imminent though their knowledge on various resistant bacterial strains was limited. Prescribers attributed ABR burden to factors such as poor prescription practices and limited ABR control measures. The prescription practices of the prescribers vary but were mostly inappropriate among the lower cadre.

**Conclusion:**

The knowledge of ABR is high among prescribers. There is however a gap in the knowledge and perception of optimal antibiotic prescription practices among prescribers. There is the need for a formal source of information on ABR to support prescriber’s antibiotic prescription practices.

**Electronic supplementary material:**

The online version of this article (doi:10.1186/s12913-017-2365-2) contains supplementary material, which is available to authorized users.

## Background

Antibiotic resistance (ABR) has become a major public health challenge in most parts of the sub-Saharan Africa including Ghana [1, 2]. Several studies have reported the escalating prevalence of ABR to cheap and available antibiotics in less developed countries [3–7]. For instance some studies carried out in Ghana in 2011 showed that the bacterial resistance to drugs such as chloramphenicol [8, 9], tetracycline, ampicillin and co-trimoxazole is high (>70%) [5].

The high burden of ABR has been an existing concern [10, 11] that has the potential of impeding appropriate health care [12] and reducing the success of child survival especially in developing countries [13] where infectious diseases are still high [14, 15], health systems are weak [16] and where the high levels of poverty [17] may make new efficacious antibiotics inaccessible to many patients. If unattended to, the effects of ABR will be similar to the consequence of chloroquine resistance observed in the late 1990s i.e. a reversal of success made to disease control [13, 18–20].

ABR can be associated with misuse of antibiotics as a result of several factors including inappropriate prescription by prescribers and misuse of antibiotics by patients [5, 12, 21]. Inappropriate prescription of antibiotics is found to be positively correlated with inappropriate use of antibiotics [21]. Interventions to curtail the increasing burden of ABR are therefore urgently needed. Many intervention programs [22] including education for prescribers and consumers (population) have been recommended to help elicit the judicious and appropriate use of antibiotics [23].

Ghana recognizes the increasing burden of ABR and is in the process of establishing policies to prevent and manage it. The role of prescribers is identified as crucial in developing interventions to control ABR [24]. The knowledge, perception, and practices of prescribers on antibiotic use and resistance was conducted to identify gaps to guide policy recommendations to curb ABR in Ghana.

## Method

### Study design

A cross-sectional survey was conducted among prescribers from both public and private facilities in the Brong Ahafo Region of Ghana using both quantitative and qualitative methods from January to March 2014.

### Study area

Brong Ahafo Region is one of the ten regions in Ghana. The region is made up of 27 districts and municipalities. It is the second largest region in Ghana and spans across a total landmass of 39,557 km^2^. Majority of the population are Akans. Christianity is the most common religion (70.8%) among the population. Illiteracy rate is high: 48.5% of persons aged 15 years and above cannot read or write in any language and about 42.0% of children are not in school [25]. The study area has one regional hospital, 27 district/municipal hospitals, 35 health centers and 102 Community Based Health Planning and Service (CHPS) compounds. These health facilities are classified into levels A, B and C. Level A is a CHPS compound, level B is a health center and level C is a hospital. Level C facilities serve as referral centers for the levels A and B facilities. There are 118 Medical Doctors (MD), 387 Physician Assistants (PA), 606 general nurses and 1094 Community Health Officers (CHOs) in the region [26]. In Ghana, the training duration for community health officers, nurses, physician assistants and medical doctors is 2, 3, 4 and 6 years respectively.

### Study approach

#### Quantitative approach

##### Sampling procedure

Medical doctors, physician assistants, nurses and community health officers were recruited to participate in the study. In each health facility, a list of medical doctors, physician assistants, nurses, and community health officers was obtained. The names of all prescribers at each health facility was written on paper and concealed by folding. The prescribers interviewed were randomly selected using a lottery method. The prescribers whose names were selected participated in the study. In cases where the number of prescribers present were equal to or less than the number required, all the prescribers were interviewed.

##### Quantitative data collection tool

All selected respondents were consented and interviewed by trained research officers, using pretested close ended questionnaires. A two paged tool with 38 questions was used to collect the quantitative data. Questions were asked to explore the knowledge of prescribers on rational antibiotic use. Prescribers responses to disease symptoms and hypothetical case scenario management was also used as a proxy to assess their knowledge on antibiotic use. The data collection instruments were pre-tested among the different category of study participants (3 medical doctors, 2 physician assistants, 5 nurses and 2 community health officers) whose data were not included in the final analysis. The questions and approach of data collection was modeled on the WHO’s standard methodology for investigating drug use in health facilities [27].

#### Qualitative approach

##### In-depth interviews

In- depth interviews were conducted among a subset of prescribers until there was no new emerging issues. The moderator was guided by a two paged tool with open ended questions. The in-depth interview tool was used to explore the perception of prescribers on ABR and patient demand for antibiotics.

The interviews were conducted in English. The moderator sought for the respondent’s consent to record and take notes on all discussions and assured respondent of confidentiality, anonymity and privacy. All recorded interviews were transcribed verbatim. Interviews were conducted between 1 and 2 h among the doctors and physician assistants.

##### Sample size calculation

The sample size was calculated using *STATA version 12 (StataCorp, College Station, Texas 77,845 USA)* It was assumed that about 70.0% of prescribers would know about ABR. At a power of 80 and 95% confidence level, a minimum of 374 prescribers was required to estimate the knowledge of ABR among prescribers in the study area assuming a worst estimate of not greater than 63.0%.

##### Data management

Completed quantitative questionnaires were checked for completeness and consistency prior to further data management at the Kintampo Health Research Centre Computer Laboratory. Data was double entered into a password-protected Microsoft FoxPro version 9.0 database. In order to protect the integrity of participants’ responses from interviews and survey questionnaires, all data related to the person was de-identified. Qualitative data was transcribed verbatim and coded electronic data are stored in password-protected computers. Data in hard copies, including survey questionnaires have been kept in locked boxes/file cabinets. Only members of the study team have access to project data. All data is reported as anonymous without making reference to specific individuals.

##### Data analysis

STATA version 11 was used to analyze data from the survey. Simple proportions have been used to describe categorical and numerical data respectively. The Fisher’s exact was performed to test for significant association between knowledge level and category of prescribers.

In the qualitative analysis, a coding list was generated based on common themes that arose from the interviews. At least two research staff independently reviewed the data, to formulate preliminary coding and to ensure inter coder reliability and data quality. Established codes were applied to all transcripts for final analysis. QSR Nvivo version 10 qualitative software was used for the qualitative data management. Transcripts were reviewed for thematic content. The findings are presented using quotes from interviews to illustrate the major themes.

## Results

A total of three hundred and seventy nine prescribers participated in the quantitative study and a subset of 33 participated in in-depth interviews. Majority (50.0%) of the prescribers interviewed were nurses (Table 1). Thirteen percent of prescribers had practiced for more than 10 years (Table 1). Most (51.0%) of the prescribers were located in Level C facility type (Hospitals) (Table 1).

**Table 1:** Profile and Length of Service of prescribers (*N* = 379)

Variable	n (%)
Sex	
Male	157 (41.0)
Female	222 (59.0)
Profession/type of respondent	
Doctor	51 (13.0)
Physician assistant	69 (19.0)
Nurse	188 (50.0)
Community Health Officer	71 (18.0)
Length of service	
0-1 year	77 (20)
2–4 years	181 (48)
5–10 years	73 (19)
More than 10 years	48 (13)
Number of years working at present facility	
0–1 year	148 (39)
2–4 years	155 (41)
5–10 years	60 (16)
More than 10 years	16 (4)
Facility type	
Level A (CHPS)	42 (11)
Level B (Health centre/Clinics)	144 (38)
Level C (Hospitals)	193 (51)

### Knowledge of ABR and use among prescribers

About 81.8% of all prescribers agreed that the antibiotics that are currently used may not be effective in future. The proportion of prescribers who agreed to this was significantly highest among doctors (96.1%) compared with community health officers (CHOs) (69.0%) *p* < 0.01 (Table 2a).

**Table 2:** Knowledge of ABR and antibiotic use among health professionals

Variables	Doctor (*n* = 51)	Physician assistant (*n* = 69)	Nurse (*n* = 188)	CHO (*n* = 71)	Total (*N* = 379)	*P* value
Possibility of antibiotics to stop working in future						
Agree	49 (96.1)	64 (92.8)	148 (78.7)	49 (69.0)	310 (81.8)	<0.01
Disagree	2 (3.9)	5 (7.2)	39 (20.8)	18 (25.4)	64 (16.9)	
Don’t know	0 (00)	0 (00)	1 (0.5)	4 (5.6)	5 (1.3)	
Antibiotics are effective in bacterial infection management						
Agree	51 (100)	67 (97.1)	180 (95.7)	69 (97.2)	367 (96.9)	0.70
Disagree	0 (00)	2 (2.9)	7 (3.8)	1 (1.4)	10(2.6)	
Don’t know	0 (00)	0 (00)	1 (0.5)	1 (1.4)	2 (0.5)	
Antibiotics are effective in viral infection management						
Agree	0 (00)	4 (5.8)	63 (33.5)	22 (40.0)	89 (23.5)	<0.01
Disagree	51 (100)	64 (92.8)	123 (65.4)	47 (66.2)	285 (75.2)	
Don’t Know	0 (00)	1 (1.4)	2 (1.1)	2 (2.8)	5 (1.3)	
Antibiotics are effective in protozoal infection management						
Agree	15 (29.4)	20 (29.0)	96 (51.1)	21 (29.6)	152 (40.1)	<0.01
Disagree	35 (68.6)	48 (69.6)	68 (36.2)	35 (49.3)	186 (49.1)	
Don’t know	1 (2.0)	1 (1.4)	24 (12.8)	15 (21.1)	41 (10.8)	
Antibiotics are effective in fungal infection management						
Agree	11 (21.6)	20 (29.0)	114 (60.6)	48 (67.6)	193 (51.0)	<0.01
Disagree	40 (78.4)	49 (71.0)	67 (35.7)	20 (28.2)	176 (46.4)	
Don’t Know	0 (00)	0 (00)	7 (3.7)	3 (4.2)	10 (2.6)	
Antibiotics are effective in managing common cold						
Agree	5 (9.8)	10 (14.5)	92 (48.9)	40 (56.3)	147 (38.8)	< 0.01
Disagree	46 (90.2)	57 (82.6)	91 (48.4)	31 (43.7)	225 (59.4)	
Don’t Know	0 (00)	2 (2.9)	5 (2.7)	0 (00)	7 (1.8)	
Antibiotics help patients to recover faster when added to malaria treatment						
Agree	2 (3.9)	3 (4.3)	42 (22.3)	27 (38.0)	74 (19.5)	< 0.01
Disagree	49 (96.1)	66 (95.7)	136 (72.3)	37 (52.1)	288 (76.0)	
Don’t know	0 (00)	0 (00)	10 (5.3)	7 (9.9)	17 (4.5)	
Antibiotics should be prescribed before lab tests are done						
Agree	12 (23.5)	5 (7.2)	19 (10.1)	12 (16.9)	48 (12.7)	0.03
Disagree	38 (74.5)	64 (92.8)	166 (88.3)	56 (78.9)	324 (85.5)	
Don’t know	1 (2.0)	0 (00)	3 (00)	3 (4.2)	7 (1.8)	
Very expensive antibiotics can be discontinued when patient is better						
Agree	8 (15.7)	10 (14.5)	38 (20.2)	20 (28.2)	76 (20.1)	0.15
Disagree	43 (84.3)	59 (85.5)	143 (76.1)	49 (69.0)	294 (69.0)	
Don’t Know	0 (00)	0 (00)	7 (3.7)	2 (2.8)	9 (2.4)	
Antibiotics use might lead to dangerous allergies which could cause death						
Agree	45 (88.8)	55 (79.7)	121 (64.4)	41 (57.7)	262 (69.1)	< 0.01
Disagree	6 (11.8)	12 (17.4)	50 (26.6)	23 (32.4)	91 (24.0)	
Don’t Know	0 (00)	2 (2.9)	17 (9.0)	7 (9.9)	26 (6.9)	
Antibiotics will always be effective in the treatment of same infections in the future						
Agree	1 (2.0)	12 (17.4)	57 (30.3)	23 (32.4)	93 (24.5)	<0.01
Disagree	49 (96.0)	55 (79.7)	125 (66.5)	39 (54.9)	268 (70.7)	
Don’t Know	1 (2.0)	2 (2.9)	6 (3.2)	9 (12.7)	18 (4.8)	
ABR is due to the normal correct use of Antibiotics						
Agree	2 (3.9)	2 (2.9)	30 (16.0)	20 (28.2)	54 (14.2)	<0.01
Disagree	48 (94.1)	67 (97.1)	151 (80.3)	45 (63.3)	311 (82.1)	
Don’t Know	1 (2.0)	0 (00)	7 (3.7)	6 (8.5)	14 (3.7)	
ABR is due to the use of Antibiotics when not prescribed						
Agree	44 (86.3)	63 (91.3)	153 (81.4)	53 (74.6)	313 (82.6)	0.03
Disagree	7 (3.7)	5 (7.2)	30 (16.0)	11 (15.6)	53 (14.0)	
Don’t Know	0 (00)	1 (1.4)	5 (2.7)	7 (9.9)	13 (3.4)	

Nineteen percent of the prescribers agreed that antibiotics can be added to malaria prescriptions to make patients recover faster. This was significantly higher among CHOs (38.0%) compared with doctors (3.9%) *p* < 0.01*.* Compared with CHOs, a significantly high proportion of doctors agreed that the use of antibiotics could lead to dangerous allergies which could cause death (*p* < 0.01) and disagreed that antibiotics will always be effective in the treatment of same infections in the future, *p* < 0.01 (Table 2b).

### Sources of knowledge about antibiotic use and resistance

The sources of information on antibiotic use and resistance among the prescribers included email alerts from platforms they subscribe to, lectures at general meetings of professional associations, books, and GHS/FDB bulletin or circulars (Table 3). The core of their knowledge was acquired during their formal training as demonstrated in the comment below:

“Aside my knowledge from school, I also learn from the BNF- British National Formulary, it has all the medications that we prescribe so, if you want to check the dose, you just refer to it and also from our standard treatment guide and also from senior colleagues” (IDI, MD)

**Table 3:** Health professionals source of information on ABR and use

Source of information	Examples
Electronic media	• Mobile phone messaging e.g. Short Messaging Service (SMS) • Internet • Email alerts by subscription
Prints	• Bulletins for Ghana Health Service or Food and Drugs Authority • British National Formulary • Text books
Meetings	• Professional meetings such as annual general meeting • Workshops • Lectures organized by pharmaceutical companies • In service training • Peers and senior colleagues

There were no differences in the sources of information among the different health professionals and there was no specific channel for obtaining information on ABR as alluded to below.

“Yes because we don't have any official source of information about antibiotics and when you browse [the internet] the information is not straight forward. If we are able to get a main source of information about antibiotics it will be well” (IDI, PA)

Barriers to prescribers access to antibiotic related information is limited by lack of internet facility, lack of a uniform source of information and lack of local data on patterns of ABR.

### Perception of the burden of ABR

Generally, the prescribers perceived ABR as a very crucial issue that needs to be addressed. Their perception of ABR burden was mainly informed by its impact on *Multidrug Resistant Mycobacterium tuberculosis* control and to a less extent about the control of other infections such as *Methicillin Resistant Staphylococcus aureus*. The prescribers perceived ABR could have serious consequences on mortality trends and health expenditure as demonstrated below:

“That is the most dangerous thing that can happen to mankind. We may move to the last limit where we cannot get any antibiotics to treat certain diseases. Talking about antibiotics, if you look at treatment of TB, which is a bacterium. We are strict because it is a combination course. If you joke and one of them develops a resistance, the other three cannot do anything. And to get another drug that will work like the former one doesn’t take 1 day. It takes a period. And the cost of it is also another thing…. So we need to be careful with antibiotics especially if mankind wants to live long on this earth” (IDI, PA)

### Perception of causes of ABR

Prescribers perception of causes of ABR were several. Their perceptions include antibiotic over prescription, irrational prescription of antibiotics, patient non-compliance to medications, unregulated dispensing of antibiotics leading to over the counter purchases. During the in-depth interviews the prescribers shared their perceptions regarding ABR from different perspectives.

“...Most of the hospitals also don’t have machines for culture sensitivity to actually isolate the real bacteria that is causing the disease. So just prescribing any form of antibiotic may increase the resistance. (IDI, PA)

“…antibiotic resistance is possible because in Ghana .., you can go to any drug store and buy any antibiotics without any prescription or these days we don’t know the source of medication that we are getting whether they are the correct or the fake ones. The efficacy level is questionable.” (IDI, MD)

However, health facility preventive measures such as hand washing, use of cleaning solutions, and sterilization were rarely mentioned by the prescribers.

### Practices of antibiotic use and health worker’s rational in selecting antibiotics

To learn about prescription practices, Upper Respiratory Tract Infections (URTI) was presented as case scenario to prescribers.

Most of prescribers indicated that they diagnose URTI using the patient’s complaints and physical examination and to a less extent laboratory confirmation of the causative agents. URTIs are often treated presumptively with antibiotics of varying types depending on the disease progression or severity at presentation. In some cases, second and third generation antibiotics are primarily used. Not all prescribers adhere to the Ghana National Treatment Guidelines in their choice of antibiotics for treating URTI. This is illustrated by the following quotes:

“Most of the URTIs practically are viral infections and not bacteria. However, if I establish that the cause of the URTI is a bacteria then I give antibiotics. As first line treatment I give amoxicillin for the typical ones or azithromycin for the atypical ones. But if the patient have already gone for the first line treatment drug, then I give the second line treatment drugs like amoxiclav and other higher antibiotics. But for kids and toddlers I give flucloxacillin for the start because the cause is usually gram positive, staph [Staphyloccocus] and strep.[Streptococcus] infection” (IDI, MD)

Prescribers rationale for their choice of antibiotics in treating patients is also influenced other factors such as patient’s ability to pay for the antibiotic, antibiotic availability patient drug history, allowable restrictions of antibiotic at the level of the facility, allowable antibiotics on insurance protocols, feedback on recovery due to treatment, patient age, medical history of the patient, and clinician wanting to impress patient. A prescriber had this to say:

“The number one reason is the insurance. You always want to prescribe what the patients will get in your facility; the second factor is affordability of the patient, if the patient can afford some of the high ones you can prescribe for them and the third factor will be previous drug history, what has the patient taken before coming to you. You don’t want to continue on the same line. The other factor will be patient choice, sometimes you have to agree with the patient” (IDI, MD)

The prescribers shared different experiences on patient’s demand for antibiotics. Prescribers do not mostly consider patients expectations in the prescription of antibiotics. However, patients who are able demonstrate why they should be prescribed a particular antibiotic may receive what they ask for. Also, patients who may have had a bad experience with a particular antibiotic may not accept the same the antibiotic if it is prescribed for them. Patients who are educated demand specific antibiotics but the less educated ones accept whatever is prescribed for them.

Prescribers also hold the perception that they know the best drugs for the patients’ illness.

“…. No! As a clinician no! If the patient comes to me with an expectation, then it is my job to explain to the patient… in certain cases it maybe what you wanted to prescribe then it makes your work easier but if it is not, I will take my time and educate the patient on what I think is best for the patient” (IDI, MD)

“You will have that problem of being influenced by patients who are educated and also the health staff themselves on the UTI (urinary tract infections), they will request specifically for some type of antibiotic but for the general population, they rely on what we prescribe for them. ” (IDI, MD)

### Knowledge of health facility infection control committee and drug or therapeutic management committees

Knowledge on the availability of an infection control committee was assessed among the prescribers. About 47.0% (178/379) of prescribers indicated non -availability of infection control committees in their facilities. Only 16.4% of prescribers reported of functional Drug Therapeutics Committee (DTC) in their facility. There was a significant difference in prescribers knowledge of a functional DTC in their facility (19.6% doctors, 20.3% PAs, 18.1% nurses and 5.6% CHOs; *p* < 0.01).

## Discussion

Prescribers are an important group of persons in the control of ABR. They are most likely to prevent ABR in their practice and advice patients on ABR if they are themselves aware of ABR. In this study, the knowledge and perception of antibiotic resistance among prescribers in the Brong Ahafo Region of Ghana was assessed. Most cadres of prescribers interviewed were knowledgeable about ABR. Most prescribers knew that the antibiotics we are using today can be ineffective in the future. There was however a significant gap in the knowledge of antibiotic use among the health professionals especially among the lower cadre of staff. It is important that this gap is bridged as the lack of knowledge of appropriate antibiotic use is likely to lead to inappropriate antibiotic stewardship and subsequently lead to antibiotic resistance. An intervention to improve antibiotic knowledge and enhance antibiotic stewardship especially among the lower cadre of prescribers is very crucial because they form the majority of prescribers in health facilities and are the frontline professionals in healthcare. In Ghana, there are no systematic programs to monitor antibiotic prescription practices among prescribers [2] and the results of this study will support the call for an urgent action against ABR in Ghana [5, 28].

Several varying factors such as existing health care systems influence antibiotic use and misuse in various parts of the world [29, 30]**.** The absence of diagnostic test at health facilities is likely to affect adherence to disease treatment protocols to prevent ABR. Almost all the prescribers interviewed knew that antibiotics are used to manage bacterial infections. However, though some prescribers knew that common cold are mostly caused by viral infections, they presumptively treat patients with antibiotics. The main reason for presumptive treatment of suspected bacterial disease is mainly due to the lack of laboratory facilities to isolate the causative organisms and also due to the syndromic approach in patient management at the community level. This practice is likely to lead to over prescription of antibiotics and subsequently bacterial resistance to available cheap antibiotics and poor management of other nonbacterial diseases. Rapid diagnostic test that could differentiate between bacterial and other infections will be important in supporting decisions to prescribe antibiotics and to reconsider syndromic approach to treatment at the lower level of care [27].

Prescribers were worried about ABR and the fact that it can be a very huge public health set back. This knowledge of the potential problem of ABR creates the opportunity for adequate health education among all cadres of prescribers about ABR.

Our finding that amoxicillin is the first line presumptively prescribed antibiotic is similar to reports of other studies that indicates that the first generation of penicillin is mostly prescribed [31, 32]. There is increasing evidence that a number of gram-negative organisms are developing resistance to amoxicillin and the frequent presumptive use of it may have a role in the emerging resistance [33, 34].

In this study, prescribers’ prescriptions were sometimes influenced by patient’s request. This finding is similar to that reported by Rodrigues et al. [35] and Abera et al. [36] In their studies, patients’ influence on prescribers to prescribe specific antibiotics was identified as one of the main factors for excessive antibiotic prescription. Though patient involvement in disease management is important to enhance disease cure, it is crucial that prescribers prescribe antibiotics based on scientific and medical basis. Prescribers rationale for their choice of antibiotics should therefore be explained to patients to enhance drug compliance.

In this study, there was no knowledge of an established ABR surveillance system where prescribers can source for information on ABR patterns in areas where they work. This is not surprising as Ghana has no formal ABR surveillance system as occurs in all other countries in the World Health Organisation (WHO) African region. [37] It is important that a national or regional ABR surveillance system such as European Center for Diseases Antimicrobial Resistance Surveillance Network [38, 39] be established to regularly update and disseminate ABR data to all health workers through effective platforms such as email or other electronic alerts, news bulletins, or through memos to all health facilities.

The role of hospital infection prevention protocols are important to prevention of bacterial resistant strains such as *Methicilin Resistant Staploccous aureus* (MRSA) [40]. In this study, the knowledge of current drug resistant strains of bacteria apart from *Multidrug Resistant Mycobacterium Tuberculosis* was limited. Specifically, the knowledge of extended-spectrum beta-lactamases producing *Enterobacteriaceae* was limited though there was an outbreak in a major hospitals in Ghana [28] and reports of other resistant bacterial stains [41, 42]. In discussing causes of drug resistance, hospital infection control was also least mentioned as potential cause of drug resistance though the Ministry of Health of Ghana has an established policy on infection control [40]. This is not surprising as less than 50% of respondents knew about their institutions infection control protocols. A functional Drug and Therapeutic Committee (DTC) in health facilities is important in ensuring rational drug prescriptions. In this study, less than 20% of respondents indicated that there was an existing functional DTC [43]. There is the need to strengthen infections control policies and DTC as institutional steps in preventing ABR.

## Conclusion

The knowledge of ABR is high among prescribers. There is however a gap in the knowledge and perception of optimal antibiotic prescription practices among health professionals. There is the need for a formal source of information of ABR to support prescriber’s antibiotic prescription practices.

## Additional file

Additional file 1: Study materials. (PDF 186 kb)

## Abbreviations

ABR: Antibiotic resistance
CHOs: Community health officers
CHPS: Community based health program and service
DTC: Drug therapeutic committee
FDB: Food and drugs board
GHS: Ghana Health Service
IDI: In depth interviews
MD: Medical doctor
PA: Physician assistant
URTI: Upper respiratory tract infection
UTI: Urinary tract infection
WHO: World Health Organisation
