# Supplementary material for: Knowledge of antibiotic resistance and antibiotic prescription practices among prescribers in the Brong Ahafo Region of Ghana; a cross-sectional study
Source: BMC Health Serv Res. 2017 Jun 20;17:422. doi: 10.1186/s12913-017-2365-2 (PMC5477684; doi:10.1186/s12913-017-2365-2)
Supplement: Additional file 1: — Study materials. (PDF 186 kb) [file 12913_2017_2365_MOESM1_ESM.pdf]

KINTAMPO HEALTH RESEARCH CENTRE<br>ANTIBIOTIC RESISTANCE KAPP STUDY 13th Jan, 2014<br>PRESCRIBERS FORM	Batch No.	Form No.

This form should be completed for identified health professionals. (Doctors, Nurses, Pharmacists, dispensers, CHOs)

We are interested in knowing more about antibiotic use and antibiotic resistance in this health facility; your knowledge and how you manage it.

We are also interested in any problems or concerns you might have about antibiotics use; and in the treatment of Urinary Tract Infections (UTIs).

We would appreciate it if you could spend about 30 minutes with us discussing the topic.
I hope this is okay with you.
Please do you have any questions?

BASIC INFORMATION

SECTION A BASIC INFORMATION

1. District Name….		DNAM
2. District Code		DCOD
3. Facility Name		FNAM
4. Facility code………………………………………………………		FCOD
5. Date of visit (DD/MM/YYYY)		DVST
6. Staff code		SCODE
7. Profession/Type of Respondent...	1. Doctor / 2. Physician Assistant / 3. Medical Assistant / 4. Nurse / 5.CHOs	PRO
8. Sex…………………………………………………………………..	1. Male / 2.Female	SEX
9. Length of service(write 00 if less than a year)		LOS
10. Number of years working at present health facility		YRS

SECTION B GENERAL KNOWLEDGE

11. It is possible for the Antibiotics we are using today to stop working properly in the future……………………	1. Agree	2. Disagree	8. Don't know	ANT
12. Antibiotics are effective in managing bacterial infections…………………………………………………..	1. Agree	2. Disagree	8. Don't know	ANTE
13. Antibiotics are effective in managing viral infections	1. Agree	2. Disagree	8. Don't know	ATEM
14. Antibiotics are effective in managing protozoal Infections…………………………………………………..	1. Agree	2. Disagree	8. Don't know	ANTT
15. Antibiotics are effective in managing fungal infections………………………………………………	1. Agree	2. Disagree	8. Don't know	ANTF

SECTION C ANTIBIOTIC USE

16. Antibiotics are effective in managing sore throat to prevent patients from getting worse…………………………………….	1. Agree	2. Disagree	8. Don't know	SORT

17. Antibiotics are effective in managing common cold to prevent

patients from getting worse………………………………………..	1. Agree	2. Disagree	8. Don't know	COLD
18. Antibiotics are effective in managing very ill patients to prevent patients from getting worse……………………………..	1. Agree	2. Disagree	8. Don't know	ILL
19. Antibiotics are effective in managing cough……………....	1. Agree	2. Disagree	8. Don't know	CUGH
20. Antibiotics are effective in managing nasal congestion….	1. Agree	2. Disagree	8. Don't know	NASL
21. Antibiotics are effective in managing stomach ache……	1. Agree	2. Disagree	8. Don't know	STCH
22. Antibiotics are to be given to **all patients** with a fever……	1. Agree	2. Disagree	8. Don't know	FEVR
23. Antibiotics should be prescribed before lab tests are done	1. Agree	2. Disagree	8. Don't know	LABT
24. Antibiotics help patients to recover faster when added to Malaria treatment………………………………………………	1. Agree	2. Disagree	8. Don't know	MATR
25. Antibiotics are good for **all** patients who have diarrhea……	1. Agree	2. Disagree	8. Don't know	DIARH
26. Very expensive antibiotics should be stopped as soon as the patient is better……………………………………..	1. Agree	2. Disagree	8. Don't know	BETR

SECTION D ANTIBIOTIC SAFETY

27. Use of antibiotics might lead to dangerous Allergies which could lead to death…………………………	1. Agree	2. Disagree	8. Don't know	DETH
28. Antibiotics are safe in pregnancy………………………	1. Agree	2. Disagree	8. Don't know	PREG
29. Antibiotics are safe in breastfeeding mothers…………	1. Agree	2. Disagree	8. Don't know	MTRS
30. Antibiotics are safe in infants……………………………..	1. Agree	2. Disagree	8. Don't know	INFTS
31. Antibiotics are safe in children under 5 years……………	1. Agree	2. Disagree	8. Don't know	CHDN

SECTION E. ANTIBIOTIC RESISTANCE

32. An antibiotic will always be effective in the treatment of the same infection in the future…………………	1. Agree	2. Disagree	8. Don't know	ALFC
33. Antibiotic resistance is due to the normal correct use of antibiotics………………………………………………………	1. Agree	2. Disagree	8. Don't know	NOUS
34. Antibiotic resistance is due to using antibiotics when they are not indicated……………………………………..	1. Agree	2. Disagree	8. Don't know	NOTN
35. In a case of UTI due to Staph. Aureus in a 25 year old, is it appropriate to prescribe ciprofloxacin?....................	1. Agree	2. Disagree	8. Don't know	UTIS

SECTION F HEALTH FACILITY INFECTIONS CONTROL

36. Does your Facility have an infections control committee?...	1.Yes	2.No	8.NK	INCON

If answer to Q. 35 is no cross out Q. 36 and Q. 37

37. Does your facility have a functional Drug Therapeutic Committee (DTC)?...	1.Yes	2.No	8.NK	DTC

38. Do you do Drug Therapeutic Committee (DTCs) activities in this health facility?..........…………	1.Yes	2.No	8. Don't know	9. NA	DTHF

Form checked by…………………………………….. | | | CHEK

KINTAMPO HEALTH RESEARCH CENTER. ANTIBIOTIC RESISTANCE KAPP STUDY (PATIENT'S FORM) DATE: 13/01/2014 VERSION 3	FORM NO.	BATCH NO.

This form should be completed for identified patients and caregivers.
We are interested in knowing more about antibiotic use and antibiotic resistance among patients and caregivers.
We are also interested in any problems or concerns you might have about antibiotic use.
We would appreciate it if you could spend about 30 minutes with us discussing the topic.
I hope this is okay with you.
Please do you have any questions?

A. BASIC INFORMATION

1. Study ID…… | | | | | | | STUDYID

2. Name of District……. | | DIST

3. District Code…….. | | | DISTCD

4. Facility type

1.Regional Hospital	2.District/Municipal Hospital	3.Health Center	FTYP
4.Private Hospital/Clinic	5.CHIPS Compound	6. Other sp…………….	

5. Date of Birth *(dd/mm/yyyy)*…. | | | | | | | | | DOB

6. Age *(approximately)*…….. | | | AGE

7. Sex…………. | 1.Male | 2.Female | SEX

8. Highest academic qualification

1.None	2.Primary School	3.Middle/JHS	MEDLEV
4.Technical/Commercial/SHS	5.Post-middle training-Teachers, Secretarial, etc.		
6.Post training –Nursing, Teachers, Polytechnic, etc.		7.University	

9. Date visit *(dd/mm/yyyy)*….. | | | | | | | | | DATVST

10. Staff Code…………….. | | | CODE

11. Do you have a valid NHIS card?.................. | 1.Yes | 2.No | NHIS

12. If yes, did you use it for this visit?(NA if 11 is 2.No | 1.Yes | 2.No | 9.NA | USNHIS

SECTION B. PATIENT KNOWLEDGE

13. What is the main complaint for which you have come to seek treatment?

Headache	1.Yes	2.No	HEAD
Stomachache	1.Yes	2.No	STOM
Diarrhea	1.Yes	2.No	DIAR
Fever	1.Yes	2.No	FEVE
Cough	1.Yes	2.No	COUGH

Lower abdominal pain	1.Yes	2.No	LWAP
Chest pains	1.Yes	2.No	CHPA
Waist Pain	1.Yes	2.No	WAPA
Common cold	1.Yes	2.No	COCO
Eye infection	1.Yes	2.No	EYINF
Ear infection	1.Yes	2.No	EARINF
Bodily pains	1.Yes	2.No	BODPA
Other	1.Yes	2.No	OTHER
Other (sp).			OTHSP

14. Did you use any medication for this compliant(s) before coming to the health facility? | 1.Yes | 2.No | USMED

15. If yes, what is/are the name(s) of the medication(s) used? (Please cross out if 3.1 is No)

Paracetamol	1.Yes	2.No	PARA
Diclofenac	1.Yes	2.No	DICL
Brufen	1.Yes	2.No	BRUF
Tricillicate	1.Yes	2.No	CIPR
Tetracycline	1.Yes	2.No	FLUC
Procold	1.Yes	2.No	FLUX
Ciprofloxacin	1.Yes	2.No	AMXC
Flagyl	1.Yes	2.No	FLGY
Amoksiklav	1.Yes	2.No	AMOX
Cotrimazole	1.Yes	2.No	COTR
Artesunate Amodiaquine	1.Yes	2.No	AA
Artemether Lumefantrine	1.Yes	2.No	AL
Coartem	1.Yes	2.No	COT
Other	1.Yes	2.No	OTHER
Otherspecify….	1.Yes	2.No	OTHSP
Other 1	1.Yes	2.No	OTH1
Other1 specify….	1.Yes	2.No	OTH1SP

16.Did the Doctor ask you to do any lab tests at this visit? | 1.Yes | 2.No | DOCT

17.If yes, which one(s)?

Blood	1.Yes	2.No	BLOOD
Urine	1.Yes	2.No	URINE
Stool	1.Yes	2.No	STOOL
Sputum	1.Yes	2.No	SPUTUM
Other	1.Yes	2.No	OTHERT
Other spe			OTHTSPE
NA	1.Yes	2.No	NA

18. Did the doctor tell you the name of your disease after seeing him? | 1.Yes | 2.No | DSNAM

19. If yes, what is/are the name(s)?

Disease Name1		DIS1
Disease Name2		DIS2
Disease Name3		DIS3

20.Did the doctor tell you the names of the drugs he is preaching for you? | 1.Yes | 2.No | DRUGS

21.If yes, what is/are the name(s) of the drug(s) (Please cross out 3.4.1 and 3.4.2 if 3.4 is No)

Paracetamol	1.Yes	2.No	DOC1
Diclofenac	1.Yes	2.No	DOC2
Brufen	1.Yes	2.No	DOC3
Ciprofloxacin	1.Yes	2.No	DOC4
Fluconazole	1.Yes	2.No	DOC5
Flucloxaclin	1.Yes	2.No	DOC6
Amoksiklav	1.Yes	2.No	DOC7
Flagyl	1.Yes	2.No	DOC8
Amoxicline	1.Yes	2.No	DOC9
Cotrimazole	1.Yes	2.No	DOC10
Artesunate Amodiaquine	1.Yes	2.No	DOC11
Artesunate Lumefantrine	1.Yes	2.No	DOC12
Coaterm	1.Yes	2.No	DOC13
Other3	1.Yes	2.No	DOC14
Other3 (specify)….			DOC14S
Other4	1.Yes	2.No	DOC15
Other4 (specify)…			DOC15S

22. Kindly ask to observe and circle all drugs that were given to patient.

Paracetamol	1.Yes	2.No	OBS1
Diclofenac	1.Yes	2.No	OBS2
Brufen	1.Yes	2.No	OBS3
Ciprofloxacin	1.Yes	2.No	OBS4
Fluconazole	1.Yes	2.No	OBS5
Flucloxaclin	1.Yes	2.No	OBS6
Amoksiklav	1.Yes	2.No	OBS7
Flagyl	1.Yes	2.No	OBS8
Amoxicline	1.Yes	2.No	OBS9
Cotrimazole	1.Yes	2.No	OBS10
Artesunate Amodiaquine	1.Yes	2.No	OBS11
Artesunate Lumefantrine	1.Yes	2.No	OBS12
Coaterm	1.Yes	2.No	OBS13
Other1	1.Yes	2.No	OBS14
Other1 (specify)….	1.Yes	2.No	OBS15
Other2	1.Yes	2.No	OBS16
Other2 (specify)…	1.Yes	2.No	OBS17

23.Did the Doctor prescribe any drug that you have to buy outside this facility?	1.Yes	2.No	OUTS

24.If yes, what is/are the names of the drug(s)?

Paracetamol	1.Yes	2.No	PARA2
Diclofenac	1.Yes	2.No	DICLO2
Brufen	1.Yes	2.No	BRUF2
Ciprofloxacin	1.Yes	2.No	CIPRO2
Fluconazole	1.Yes	2.No	FLUCN2
Flucloxacllin	1.Yes	2.No	FLUCX2
Amoksiklav	1.Yes	2.No	AMXCL2
Flagyl	1.Yes	2.No	FLGY2
Amoxicline	1.Yes	2.No	AMOX2
Cotrimazole	1.Yes	2.No	COTR2
Artesunate Amodiaquine	1.Yes	2.No	AA2
Artemether Lumefantrine	1.Yes	2.No	AL2
Coaterm	1.Yes	2.No	COT2
Other2	1.Yes	2.No	OTH2

Other2(specify)			OTH2SP
Other3	1.Yes	2.No	OTH3
Other3(specify)			OTH3SP

25. Do you know what an antibiotic is? **(eg flagyl, amoxicillin, amoksiklav)** | 1.Yes | 2.No | KNAB

26. Have you ever bought antibiotics without prescription? | 1.Yes | 2.No | PRSC

27. If yes, what did you use it for? (Please cross out if patient has not bought an antibiotic without pres)

Headache	1.Yes	2.No	HEAD1
Stomachache	1.Yes	2.No	STOM1
Diarrhea	1.Yes	2.No	DIAR 1
Fever	1.Yes	2.No	FEVE1
Cough	1.Yes	2.No	COUGH1
Lower abdominal pain	1.Yes	2.No	LWAP1
Chest pains	1.Yes	2.No	CHPA1
Waist pains	1.Yes	2.No	WAPA1
Common Cold	1.Yes	2.No	COCO1
Eye infection	1.Yes	2.No	EYINF1
Ear infection	1.Yes	2.No	EARINF1
Bodily pains	1.Yes	2.No	BODPA1
Other1	1.Yes	2.No	OTHER1
Other1(specify)	1.Yes	2.No	OTH1SP

28. Did the doctor prescribe an antibiotic for you during this episode of illness? | 1.Yes | 2.No | ANTIBIO

29. How many types of antibiotics were prescribed for you?(enter 99 if no antibiotic) | | | TYPE

30. Did you ask the doctor to prescribe any of these antibiotics for you? | 1.Yes | 2.No | 9.NA | ASKFP

31. Have you ever asked a doctor/prescriber to prescribe an antibiotic for you? | 1.Yes | 2.No | DEMAND

32. For what sickness did you ask for this antibiotic to be prescribed for you?

Headache	1.Yes	2.No	HEAD2
Stomachache	1.Yes	2.No	STOMS
Diarrhea	1.Yes	2.No	DIAR2
Fever	1.Yes	2.No	FEVE2
Cough	1.Yes	2.No	COUGH2
Lower abdominal pain	1.Yes	2.No	LWAP2
Chest pains	1.Yes	2.No	CHPA2
Waist pain	1.Yes	2.No	WAPA2
Common Cold	1.Yes	2.No	COCO2
Eye infection	1.Yes	2.No	EYINF2
Ear infection	1.Yes	2.No	EARINF2
Bodily pains	1.Yes	2.No	BODPA2
Other2	1.Yes	2.No	OTHER2
Other2 (specify)			OTHERSP

33. Did you obtain all the antibiotics prescribed for you at the dispensary? | 1.Yes | 2.No | 9.NA

34. If no, which ones did you not obtain?

Antibiotic 1		NOTOB1
Antibiotic 2		NOTOB2
Antibiotic 3		NOTOB3

35. Will you accept an alternative medication from the dispenser for any prescribed antibiotics not available at the dispensary? | 1.Yes | 2.No | ALTERN

36. If No, why? (if yes, NA)

	9.NA	WHY3

37. Did the doctor here also give you a prescription for an antibiotic that you must buy outside of this facility? (please observe from prescription)

1.Yes	2.No	BUY

38. If yes, how many?

		NBUY

39. Did the dispenser here explain to you why you have to take this antibiotic?

1.Yes	2.No	WYTK

40. Did the dispenser tell you how to take the antibiotics?

1.Yes	2.No	HWTK

41. Would you please tell me how to take the antibiotics? (Look at the prescription, box, and sachet, if appropriately labeled and compare patient's answer. (Cross out if 3.13 is No)

**1st Antibiotic**		
1.Correctly stated	2.Not correctly stated	TEL1
**2nd Antibiotic**		
1.Correctly stated	2.Not correctly stated	TEL2
**3rd Antibiotic**		
1.Correctly stated	2.Not correctly stated	TEL3

SECTION D: PATIENT SATISFACTION

42. Are you satisfied with the services provided to you today? (From OPD to Dispensary)?

1.Very satisfied	2.Faily satisfied	3.Disatisfied	4.Highly Dissatisfied	SATF

43. What are the main reasons for your option in question 4.0 above?

1.Attitude of service providers	REASON1
2.Waiting time	REASON2
3.Medication	REASON3
4.Logistics,	REASON4
5. Other specify	REASON5

44. Ask and record any other comments the client has about visit to this facility today.

	OHTCM

45. Form checked by…………………….

		CHECK

KINTAMPO HEALTH RESEARCH CENTRE<br>ANTIBIOTIC RESISTANCE KAPP STUDY 14AUG13<br>FOLDER REVIEW FORM version 1.	Batch No.	Form No.

(Form should be filled for the last three hospital visits)

DEMOGRAPHIC CHARACTERISTICS

1. District Name….. | DNAM
2. District Code | DCOD
3. Facility Name | FNAM
4. Facility code……………………………………………………… | FCOD
5. Folder number………….. | FLDN
6. Age in years……………… | AGE
7. Sex………………………….. | 1. Male | 2. Female | SEX
8. Profession of consultant | 1. Doctor | 2. Physician Assistant | 3. Medical Assistant | 4. Prescribing nurse | RETYP
9. Is the client registered with NHIS | 1. Yes | 2. No | HIREG
10. Staff code…………………………………………. | CODE

Visit one

11. Date of first visit | VISIT1

12. What was the final diagnosis for the patients' visit to the health facility.

1. Malaria	1.Yes	2.No	DGNS1
2. Diarrhoea	1.Yes	2.No	DGNS2
3. Upper Respiratory Tract Infection	1.Yes	2.No	DGNS3
4. Skin diseases	1.Yes	2.No	DGNS4
5. Hypertension	1.Yes	2.No	DGNS5
6. Pneumonia	1.Yes	2.No	DGNS6
7. Anaemia	1.Yes	2.No	DGNS7
8. Intestinal worms	1.Yes	2.No	DGNS8
9. Rheumatism	1.Yes	2.No	DGNS9
10. Ear infection	1.Yes	2.No	DGNS10
11. Other specify……………………			DGNS11

13. Was laboratory test(s) requested?........................ | 1. Yes | 2. No | LABT1

If answer to Q 2.2 is No, cross out 2.3, 2.4

14. Was lab test done?.................................. | 1. Yes | 2. No | LABD1

15. If yes, what was the result?…… | LABR1 | LABR2 | LABR3

16. Which drugs were prescribed for

1.Ciproflaxacilin	1. Yes	2. No	9. NA	DRG1
2.Flagll	1. Yes	2. No	9. NA	DRG2
3.Penicillin	1. Yes	2. No	9. NA	DRG3

patient? (List all drugs in folder)

4.Amoxicillin	1. Yes	2. No	9. NA	DRG4
5.Amoxiclave	1. Yes	2. No	9. NA	DRG5
6. Other (specify)………………….	1. Yes	2. No	9. NA	DRG6
	1. Yes	2. No	9. NA	DRG7

Visit two

17. Date of first visit

									VISIT1

18. What was the final diagnosis for the patients' visit to the health facility.

12. Malaria	1.Yes	2.No	DGNS12
13. Diarrhoea	1.Yes	2.No	DGNS13
14. Upper Respiratory Tract Infection	1.Yes	2.No	DGNS14
15. Skin diseases	1.Yes	2.No	DGNS15
16. Hypertension	1.Yes	2.No	DGNS16
17. Pneumonia	1.Yes	2.No	DGNS17
18. Anaemia	1.Yes	2.No	DGNS18
19. Intestinal worms	1.Yes	2.No	DGNS19
20. Rheumatism	1.Yes	2.No	DGNS20
21. Ear infection	1.Yes	2.No	DGNS21
22. Other specify……………………	1.Yes	2.No	DGNS22

19. Was laboratory test(s) requested?......................... | 1. Yes | 2. No | LABT2

If answer to Q. 3.2 is No, cross out 3.3, 3.4

20. Was lab test done?.................................. | 1. Yes | 2. No | LABD2

21. If yes, what was the result?……

	LABR4
	LABR5
	LABR6

22.Which drugs were prescribed for patient? (List all drugs in folder)

1.Ciproflaxacilin	1. Yes	2. No	9. NA	DRG8
2.Flagll	1. Yes	2. No	9. NA	DRG9
3.Penicillin	1. Yes	2. No	9. NA	DRG10
4.Amoxicillin	1. Yes	2. No	9. NA	DRG11
5.Amoxiclave	1. Yes	2. No	9. NA	DRG12
6. Other (specify)………………….	1. Yes	2. No	9. NA	DRG13

Visit three

23. Date of first visit

									VISIT1

24. What was the final diagnosis for the patients' visit to the health.

1.Malaria	1. Yes	2. No	DGNS23
2.Diarrhoea	1. Yes	2. No	DGNS24
3.Upper Respiratory Tract Infections	1. Yes	2. No	DGNS25
4.Hypertensiom	1. Yes	2. No	DGNS26
5.Pneumonia	1. Yes	2. No	DGNS27
6.Anaemia	1. Yes	2. No	DGNS28
7.Instestinal worms	1. Yes	2. No	DGNS29

8.Rheumatism	1. Yes	2. No	DGNS30
9.Ear Infection	1. Yes	2. No	DGNS31
10.Skin diseases and ulcer	1. Yes	2. No	DGNS32

25. Was laboratory test(s) requested?......................... | 1. Yes | 2. No | LABT2

If answer to Q. 3.2 is No, cross out 3.3, 3.4

26. Was lab test done?.................................. | 1. Yes | 2. No | LABD2

27. If yes, what was the result?……

	LABR7
	LABR8
	LABR9

28. Which drugs were prescribed for patient? (List all drugs in folder)

1.Ciproflaxacilin	1. Yes	2. No	9. NA	DRG14
2.Flagll	1. Yes	2. No	9. NA	DRG15
3.Penicillin	1. Yes	2. No	9. NA	DRG16
4.Amoxicillin	1. Yes	2. No	9. NA	DRG17
5.Amoxiclave	1. Yes	2. No	9. NA	DRG18
6. Other<br><br>(spec)………………….	1. Yes	2. No	9. NA	DRG19<br>DRG19S

Form Checked by……………………………………………………..........

KINTAMPO HEALTH RESEARCH CENTER, KINTAMPO.
KNOWLEDGE, ATTITUDES, PERCEPTIONS AND PRACTICES OF HEALTH PROFFESSIONALS ON ANTIBIOTIC USE AND RESISTANCE IN GHANA STUDY

INDEPTH INTERVIEWS

Version 1.   Date 15/01/2014

Knowledge about infectious diseases in the community:

1. What are the most common infections reported by patients in this health facility?
    a. Probe: How about Urinary Tract Infections (UTI) and Upper Respiratory Tract Infections(URTI)? (If not mentioned)
    b. How common are these in relation to other common diseases or infections?

Diagnosis of UTIs

2. How do URTI and UTIs cases usually present here?
    c. Probe: What kinds of symptoms do patients usually have?
    d. How do you decide that patients presenting this way have UTIs? (Probe questions asked in history, physical examination, Labs)

3. Nature Of UTIs cases
    a. Can you describe to me the characteristics of patients who usually suffer from UTIs?
    b. Probe: Age, socio-economic status

4. Knowledge about community perception of UTIs.
    c. What do people in the community think about UTIs when they or their relative gets an attack?
    d. Probes: What are their anxieties?
    e. Do they use any medications including home medications before seeking treatment?

5. UTIs Case Management
    f. How do you usually treat UTIs?
    g. Probe: What are the common medications that you prescribe for UTIs?
    h. Are there any factors that influence your choice of antibiotics when treating UTIs?
    i. How much does a typical treatment episode cost?
    j. Are patients able to afford this cost?
    k. Are patients able to get the antibiotics they need here most of the time?

6. Prescribing
7.
    l. Probe: Are the antibiotics you frequently prescribe for UTIs usually antibiotic or brand?
    m. What are the reasons for your common preference?
    n. Does patient expectation play a role in the type of antibiotics that are prescribed?
    o. How and why do you feel this way?
    p. When you prescribe a brand name that is not available, what do you do? Evidence suggests that antibiotic prescribing is very low among prescribers. What is your opinion about this?

Diagnosis of URTIs

8. How do URTI cases usually present here?
    e. Probe: What kinds of symptoms do patients usually have?
    f. How do you decide that patients presenting this way have URTIs? (Probe questions asked in history, physical examination, Labs)

9. Nature of URTIs cases
    q. Can you describe to me the characteristics of patients who usually suffer from URTIs?
    r. Probe: Age, socio-economic status

10. Knowledge about community perception of URTIs.

s. What do people in the community think about URTIs when they or their relative gets an attack?
t. Probes: What are their anxieties?
u. Do they use any medications including home medications before seeking treatment?

11. URTIs Case Management
v. How do you usually treat URTIs?
w. Probe: What are the common medications that you prescribe for URTIs?
x. Are there any factors that influence your choice of antibiotics when treating URTIs?
y. How much does a typical treatment episode cost?
z. Are patients able to afford this cost?
aa. Are patients able to get the antibiotics they need here most of the time?

12. Prescribing
bb. What is your opinion about antibiotics resistance?
cc. Probe: Are the antibiotics you frequently prescribe for URTIs usually antibiotic or brand?
dd. What are the reasons for your common preference?
ee. Does patient expectation play a rule in the type of antibiotics that are prescribed?
ff. How and why do you feel this way?
gg. When you prescribe a brand name that is not available, what do you do? Evidence suggests that antibiotic prescribing is very low among prescribers. What is your opinion about this?

13. Therapeutics and Education
Finally, I would like to hear about how you get information about antibiotics resistance.
a. First, what do you know about the rational use of antibiotics?
b. Probe: What are some of the ways that you learn about antibiotics prescribing antibiotics?
c. Do you have difficulties in learning about new information about antibiotics?
d. Are there any suggestions you could make to remedy this situation?
e. Do you think you need further education in antibiotic prescribing?
f. What type of education do you need?
g. How would you benefit from such education?

Comments
14. Is there anything else important you can think of about treating UTIs that we have not talked about?

CLOSE: Thank health worker for his/her comments.
